# Supplementary material for: Chloramphenicol and gentamicin reduce the evolution of resistance to phage ΦX174 by suppressing a subset of E. coli LPS mutants
Source: PLoS Biol. 2025 Jan 21;23(1):e3002952. doi: 10.1371/journal.pbio.3002952 (PMC11753469; doi:10.1371/journal.pbio.3002952)
Supplement: S1 Text — Note B. Parallel evolution in the fluctuation test. Note C. Predicted mutations from the fluctuation test that occur in genes not known to be related to LPS biosynthesis, assembly, or regulation. (DOCX) [file pbio.3002952.s001.docx]

**Note A.**

*Explanation of differences in antibiotic susceptibility profiles between isogenic bacterial strains in Fig 2.*

*E. coli* C wildtype-derived, phage-resistant strains R27 and R31 are genetically identical (Romeyer Dherbey et al., 2023), yet slightly different gentamicin growth profiles were measured for these mutants (**Fig 2A**). Specifically, R27 growth is fully inhibited at 2 µg/mL gentamicin, while R31 growth is slightly above the detection threshold. R31 growth is fully inhibited at the next gentamicin concentration measured (3 µg/mL). This discrepancy could be accounted for by small variations – e.g. in bacterial growth or media preparation – if 2 µg/mL were very close to the MIC of the R27/R31 genotype. If so, we would expect to observe growth slightly under or above the detection threshold in different (biological or technical) replicates. Detection of growth would then depend on the particular threshold. Indeed, when the growth detection threshold is increased to an average grayscale value of 12 (from 8), both R27 and R31 have an MIC of 2 µg/mL for gentamicin (see **S1 Table**).

**Note B.**

## Parallel evolution in the fluctuation test

Some parallel evolution was observed in the fluctuation test; some independent isolates carry identical (loss-of-function) mutations, both within and between plating environments. For example, an 18 bp deletion (positions 74,589 - 74,606) occurs in seven mutants (1 from ΦX174-only, 1 from ΦX174+CL(1 µg/mL), 4 from ΦX174+GM(1 µg/mL), 1 from ΦX174+GM(2 µg/mL). In each of *hldD* and *waaT*, two mutations each occur in different replicates in the same environment. Notably, reductions in mutational target size do not appear to lead to higher degrees of parallelism; the most parallelism is seen in ΦX174+GM(1 µg/mL) (where there no mutational target size reduction is expected). Specifically, 1 *hldE* mutation is observed three times, 1 *hldD* mutation is observed twice, and 1 *waaG* mutation occurs four times. An alternative explanation for parallelism may be mutational bias (i.e., genomic loci that, due to specific features, are more susceptible to mutation) (Lind et al., 2019; Bertels et al., 2021).

**Note C.**

## Predicted mutations from the fluctuation test that occur in genes not known to be related to LPS biosynthesis, assembly, or regulation

Several of the phage-resistant *E. coli* C mutants from the fluctuation test carry mutations in genes that are not known to be involved in the biosynthesis, assembly, or regulation of LPS.

These include:

*dosP* (ΦX174-only environment): adjacent genes *dosP* and *dosC* collectively regulate the level of cyclic diguanylate (c-di-GMP) in the cell. C-di-GMP promotes the production of exopolysaccharides and other substances related to the extracellular matrix [1]. DosP is involved in c-di-GMP degradation, thus a loss-of-function *dosP* mutation is expected to increase c-di-GMP levels and thus promote the production of extracellular polysaccharides.

*yfcV* (ΦX174+CL(1 µg/mL)): *yfcV* encodes a fimbrial protein, which is a known virulence factor in uropathogenic *E. coli* [2]. Since this fimbrial protein can increase adhesion of bacterial cells to eukaryotic epithelial cells *in-vitro*, it may also contribute to adhesion in other environments [3].

*glpG* (ΦX174+GM(1 µg/mL)): *glpG* encodes an intra-membrane protease [4].

*yidC* (ΦX174+GM(2 µg/mL)): *yidC* is an essential gene that encodes a membrane protein insertase, which is part of the SecDFyajC-YidC holotranslocon membrane insertase complex [5]. Phages can use YidC for insertion of phage coat proteins through the membrane [6].

**Supplementary References**

1. Laventie BJ, Jenal U. Surface Sensing and Adaptation in Bacteria. Annual Review of Microbiology. 2020;74(1):735–60.

2. Spurbeck RR, Dinh PC, Walk ST, Stapleton AE, Hooton TM, Nolan LK, et al. Escherichia coli Isolates That Carry vat, fyuA, chuA, and yfcV Efficiently Colonize the Urinary Tract. Infect Immun. 2012 Dec;80(12):4115–22.

3. Korea CG, Badouraly R, Prevost MC, Ghigo JM, Beloin C. Escherichia coli K-12 possesses multiple cryptic but functional chaperone-usher fimbriae with distinct surface specificities. Environ Microbiol. 2010 Jul;12(7):1957–77.

4. Keseler IM, Gama-Castro S, Mackie A, Billington R, Bonavides-Martínez C, Caspi R, et al. The EcoCyc Database in 2021. Front Microbiol. 2021 Jul 28;12:711077.

5. Nouwen N, Driessen AJM. SecDFyajC forms a heterotetrameric complex with YidC. Mol Microbiol. 2002 Jun;44(5):1397–405.

6. Kol S, Nouwen N, Driessen AJM. Mechanisms of YidC-mediated Insertion and Assembly of Multimeric Membrane Protein Complexes*. Journal of Biological Chemistry. 2008 Nov 14;283(46):31269–73.
