# Supplementary material for: Chloramphenicol and gentamicin reduce the evolution of resistance to phage ΦX174 by suppressing a subset of E. coli LPS mutants
Source: PLoS Biol. 2025 Jan 21;23(1):e3002952. doi: 10.1371/journal.pbio.3002952 (PMC11753469; doi:10.1371/journal.pbio.3002952)
Supplement: S2 Text — The figure shows mean greyscale values for growth of different E. coli C strains combined with different chloramphenicol concentrations. There are between 1 and 5 measurements for each combination shown on the x-axis. Each measurement is the mean greyscale value across a region of 1,157.85 mm2 in a photo of an LB plate inoculated with an E. coli C strain (see Methods). The black horizontal line at a greyscale value of 37 indicates the mode of the mean background greyscale value of blank LB plates without addition of bacteria. The green horizontal line shows the strict growth cut-off of 8 (37+8) and the red horizontal line shows the lenient growth cut-off of 12 (37+12). The colours of the datapoints indicate whether the strain is predicted to possess a rough or a deep rough LPS phenotype. The data underlying this figure (greyscale values of replicates and raw images) can be found in https://doi.org/10.17617/3.PIDVUT. Fig S2. Raw mean greyscale values measured for strains grown in the presence of gentamicin. For details, see S1 Fig. Fig S3. Mean greyscale values of strains grown in the presence of chloramphenicol (A) or gentamicin (B), clustered by whether the strain is rough or deep rough. The figure shows the mean greyscale values for the growth of E. coli C at different antibiotic concentrations on LB plates for all strains shown in Fig 2. Each measurement is the mean greyscale value across a region of 1,157.85 mm2 in a photo of an LB plate inoculated with an E. coli C strain. The black horizontal line at a greyscale value of 37 indicates the mode of the mean background greyscale value, that is, of blank LB plates without addition of bacteria. The green horizontal line shows the strict growth cut-off of 8 (37+8) and the red horizontal line shows the lenient growth cut-off of 12 (37+12). The colours of the datapoints indicate whether the strain is predicted to possess a rough or a deep rough LPS phenotype. The data underlying this figure (greyscale values of replica [file pbio.3002952.s002.docx]

**Supplementary Figures**

**
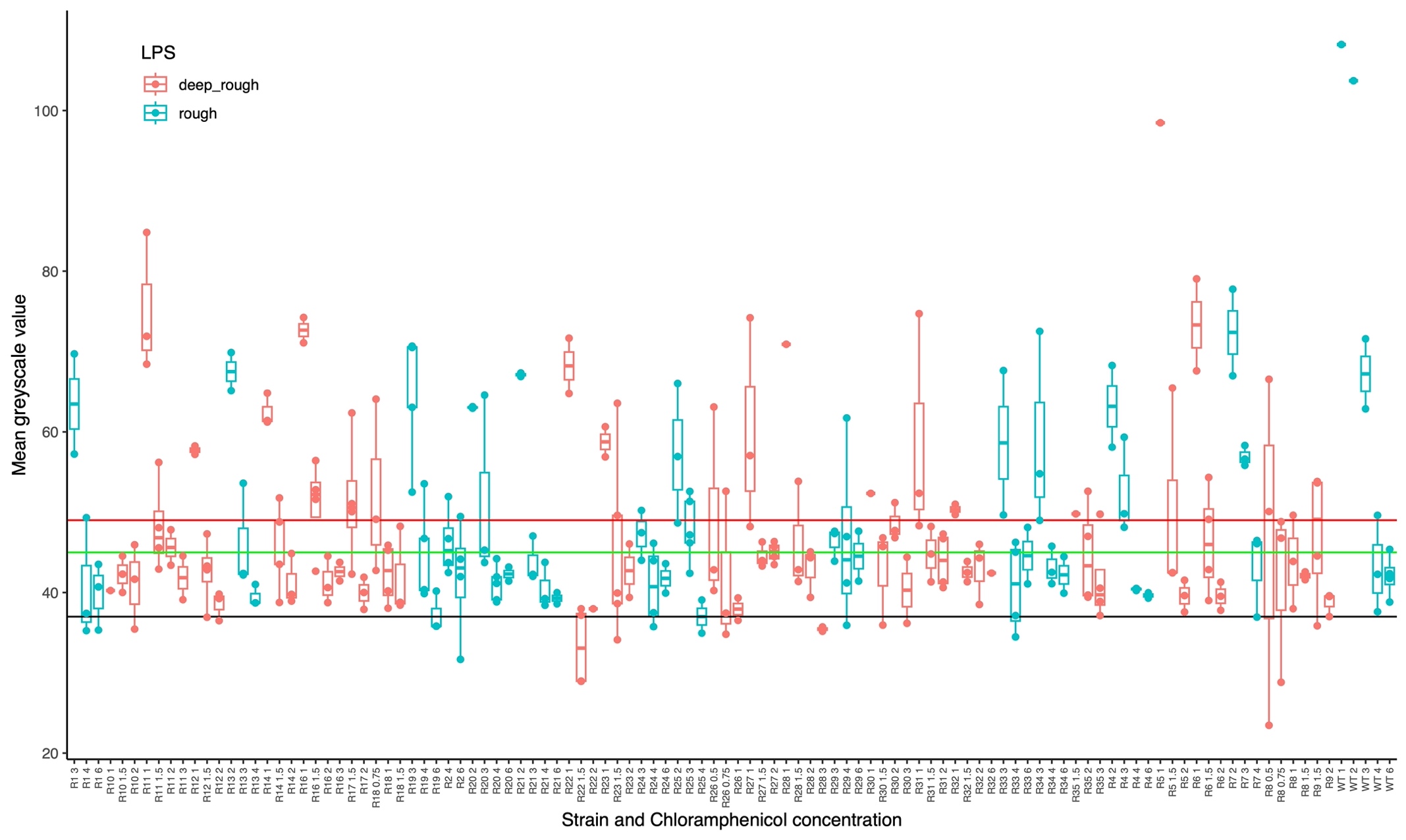
**

**Fig S1. Raw mean greyscale values measured for strains grown in the presence of chloramphenicol.** The figure shows mean greyscale values for growth of different *E. coli* C strains combined with different chloramphenicol concentrations. There are between one and five measurements for each combination shown on the x-axis. Each measurement is the mean greyscale value across a region of 1157.85 mm² in a photo of an LB plate inoculated with an *E. coli* C strain (see **Methods**). The black horizontal line at a greyscale value of 37 indicates the mode of the mean background greyscale value of blank LB plates without addition of bacteria. The green horizontal line shows the strict growth cut-off of 8 (37+8) and the red horizontal line shows the lenient growth cut-off of 12 (37+12). The colours of the datapoints indicate whether the strain is predicted to possess a rough or a deep rough LPS phenotype. The data underlying this figure (greyscale values of replicates and raw images) can be found in <https://doi.org/10.17617/3.PIDVUT>.

**
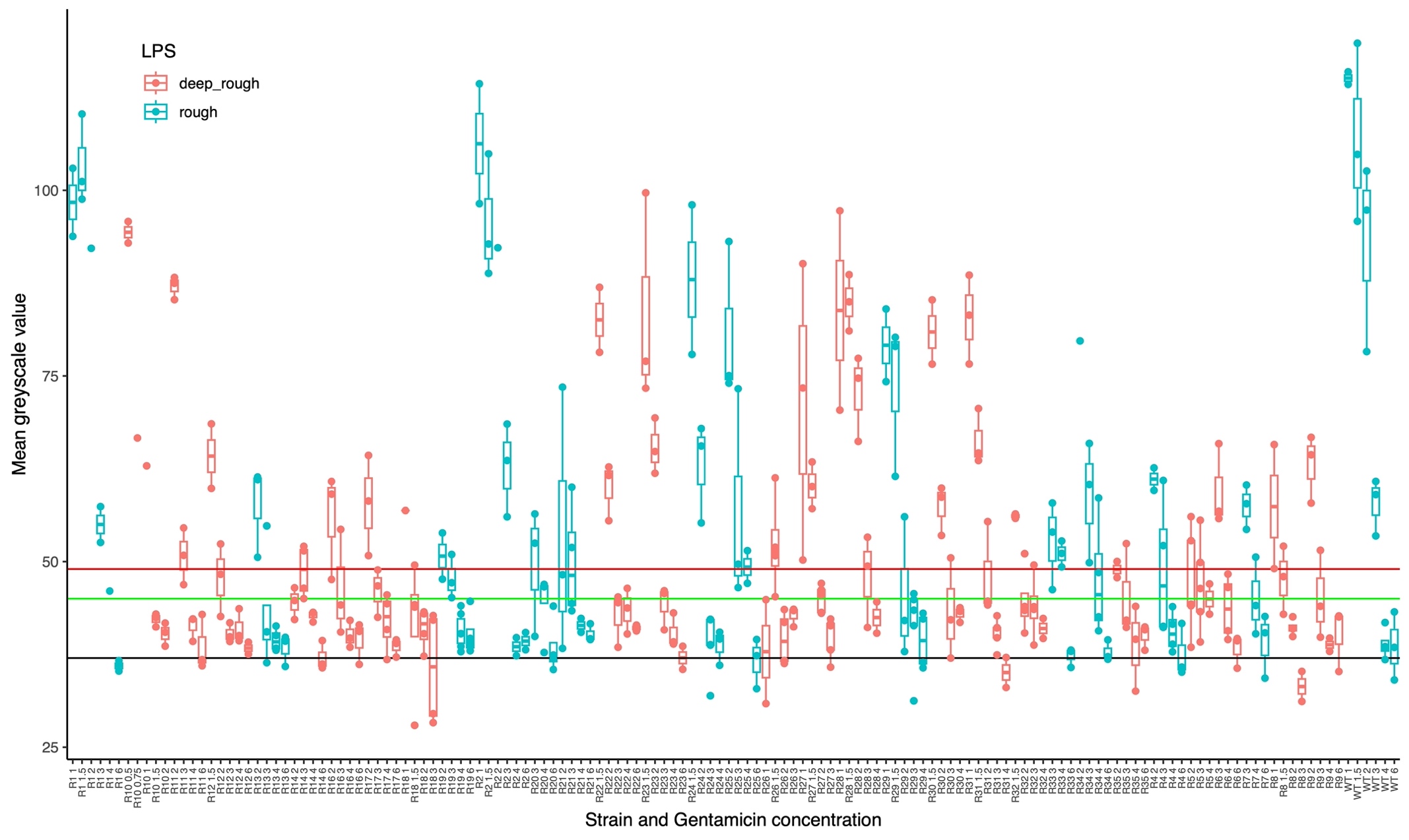
**

**Fig S2. Raw mean greyscale values measured for strains grown in the presence of gentamicin.** For details, see Fig S1.

**Fig S3. Mean greyscale values of strains grown in the presence of chloramphenicol (A) or gentamicin (B), clustered by whether the strain is rough or deep rough.** The figure shows the mean greyscale values for the growth of *E. coli* C at different antibiotic concentrations on LB plates for all strains shown in **Fig 2**. Each measurement is the mean greyscale value across a region of 1157.85 mm² in a photo of an LB plate inoculated with an *E. coli* C strain. The black horizontal line at a greyscale value of 37 indicates the mode of the mean background greyscale value, that is, of blank LB plates without addition of bacteria. The green horizontal line shows the strict growth cut-off of 8 (37+8) and the red horizontal line shows the lenient growth cut-off of 12 (37+12). The colours of the datapoints indicate whether the strain is predicted to possess a rough or a deep rough LPS phenotype. The data underlying this figure (greyscale values of replicates and raw images) can be found in <https://doi.org/10.17617/3.PIDVUT>.


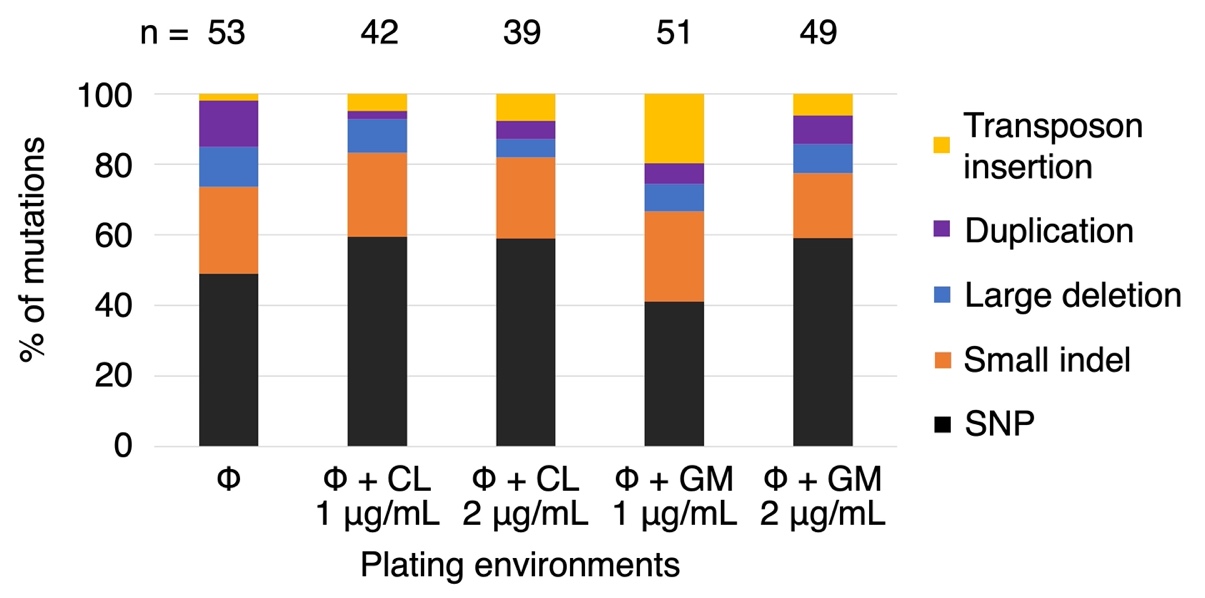


**Fig S4. Types of mutations identified across phage-resistant *E. coli* C strains isolated from the fluctuation test.** Bars show all predicted mutations grouped by mutational class (as % of total mutations “n” identified in each of the five plating environments sampled). If an isolate had two mutations, they were classified and counted separately. Insertions and deletions are further separated into four categories: (i) transposon insertion, where the inserted bases are a transposable element, (ii) duplication, where the inserted bases match the sequence preceding the mutated locus (up to 1.3 kbp), (iii) large deletion, where a region of more than 100 bp is deleted, and (iv) small indels, other insertions and deletions of <100 bp. CL = chloramphenicol, GM = gentamicin. The data underlying this figure (mutations) can be found in **S4 Table.**


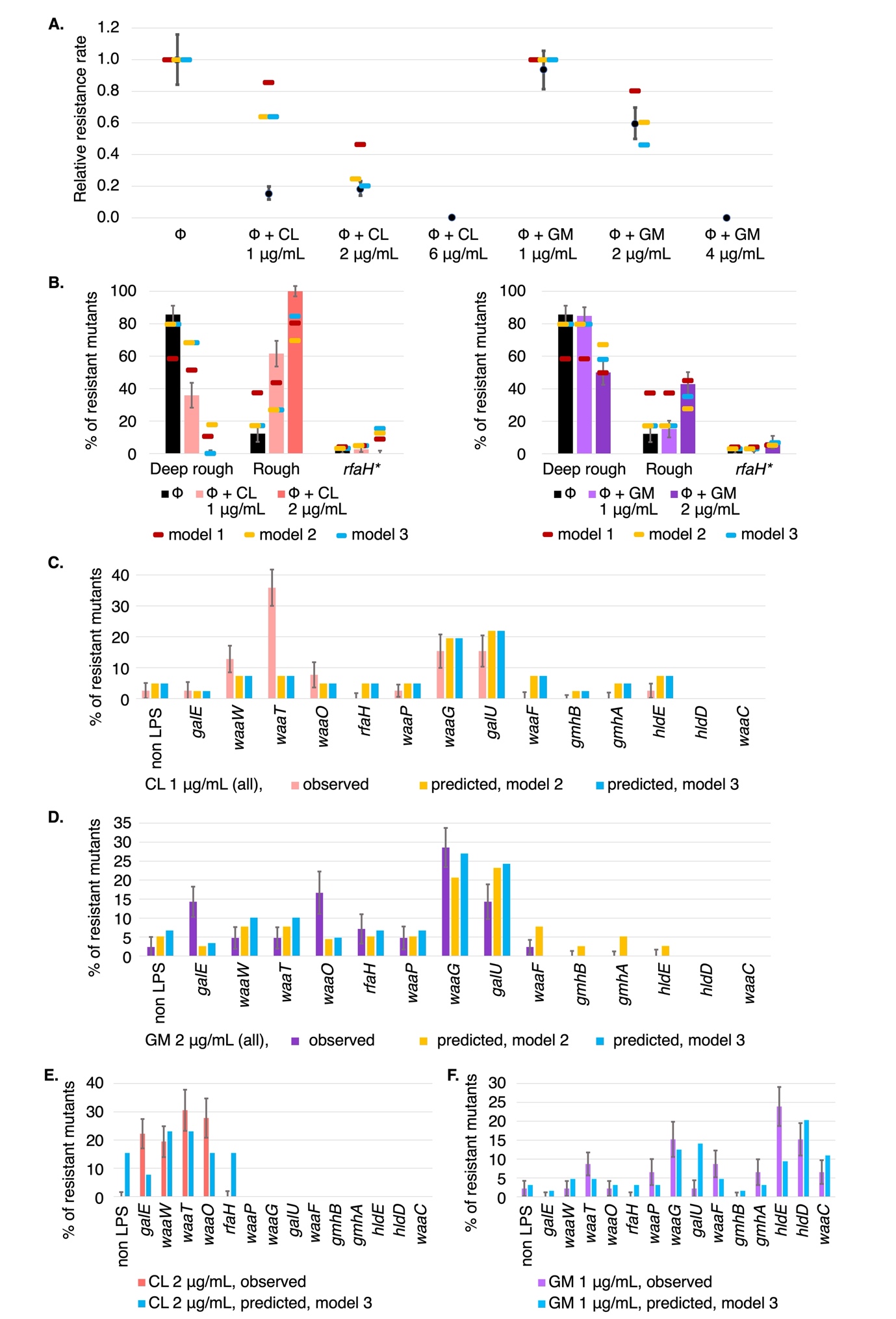


**Fig S5. Comparison of model 3 predictions with observed phage-resistance rates.** (**A**) Phage-resistance rates for phage-antibiotic combinations, relative to the phage-resistance rate in the ΦX174-only environment. Observed rates (black) are only qualitatively in line with the predicted rates from model 1 (gene length model, dark red; see **Table 1**). Rates predicted by model 2 (dark yellow; derived in **S6 Table**) are within 95 % confidence intervals for ΦX174+gentamicin environments, and are 2 % away from the 95 % confidence interval in ΦX174+chloramphenicol (2 µg/mL) environment. Phage-resistance rates for model 3 (blue, **S7 Table**) remain largely unchanged (relative to those for model 2), except for the ΦX174+gentamicin(2 µg/mL) environment (where the prediction becomes worse). (**B**) LPS phenotype distribution in phage-resistant *E. coli* C mutants isolated in the fluctuation test. Graphs show proportion of mutants with a certain LPS phenotype (values in **S5 Table**). Model 1 predictions (dark red) are qualitatively in line with predictions. In model 2 (dark yellow), LPS phenotype distribution in the ΦX174-only environment is now in line with the observations. Model 3 predictions (blue) improve slightly for ΦX174+chloramphenicol (2 µg/mL) and ΦX174+gentamicin (2 µg/mL) environments. (**C-F**) Gene-wise distribution compared to model 3 predictions (and model 2 in C-D). In ΦX174+chloramphenicol (1 µg/mL), mutations are predicted in *waaF, gmhB, gmhA* and *rfaH*, but none are observed. Similarly, in ΦX174+gentamicin (2 µg/mL), mutations in *gmhB*, *gmhA*, *hldE* are predicted but not observed. Compared to model 2, only predictions at ΦX174+chloramphenicol (2 µg/mL) (*waaP, galU*) and ΦX174+gentamicin (2 µg/mL) (*gmhB*, *gmhA*, *hldE*) change in model 3. Error bars show the standard deviation for each LPS phenotype (**B**) or each gene (**C-F**) from 100 bootstrapping iterations (resampling with replacement, see **Methods**). The data underlying this figure can be found in **S4-S7 Tables**, which include raw mutation data as well as summarised values.
