# Supplementary material for: Chloramphenicol and gentamicin reduce the evolution of resistance to phage ΦX174 by suppressing a subset of E. coli LPS mutants
Source: PLoS Biol. 2025 Jan 21;23(1):e3002952. doi: 10.1371/journal.pbio.3002952 (PMC11753469; doi:10.1371/journal.pbio.3002952)
Supplement: Attachment — Submitted filename: Reply_to_reviewers_thirdsubmission_final.docx [file pbio.3002952.s010.docx]

Dear editor and reviewers,

We are grateful for the constructive comments we have received. We have made a number of changes to the manuscript. Most significantly, we have (i) rewritten the manuscript improve flow and understanding, (ii) added analyses for the data presented in figure 2, and (iii) added a co-evolution experiment with bacteria, phages, and antibiotics in liquid culture. Below we list the changes in more detail:

1. New title: “Chloramphenicol and gentamicin reduce resistance evolution to phage ΦX174 by suppressing a subset of *E. coli* C LPS mutants”. We changed the title to include the effect of gentamicin in the combination treatments.
2. One additional coevolution experiment: We had previously shown how antibiotics affect the evolution of ΦX174 resistance in a one-day fluctuation experiment in semisolid medium. We now also include a short-term coevolution experiment where we compare bacterial growth in phage-antibiotic combinations (same as fluctuation experiment) over two transfers, using either phage or antibiotic alone as controls.

The results further support the conclusions of our fluctuation experiment. On day 1, the most influential factor for bacterial growth is whether or not phages are present. Populations with phages experience a rapid decrease in numbers, until phage resistance appears and bacterial cultures start growing again. Phage resistance appears earlier in the phage-only environment followed by phage+gentamicin and then phage+chloramphenicol. On day 2, the presence of phages is no longer the most influential factor on growth of the bacterial population. Instead, growth of the day 2 populations groups by antibiotic: cultures with no antibiotic now grow most quickly, then those with gentamicin, and finally those with chloramphenicol (in each case, regardless of phage presence or absence). These results match the fluctuation experiment, in which the highest number of mutational routes to phage-resistance are accessible in the absence of either antibiotic, with the presence of gentamicin and chloramphenicol leading to progressively fewer available mutations.

3. We added **Figure 2B**, which shows antibiotic MIC values of phage-resistant *E. coli* C strains, and compares MICs of deep rough and rough *E. coli* C strains. To address a reviewer concern, we now also provide figures showing the raw growth measurements for each replicate in **Supplementary Figures S1-S3**.
4. We edited the manuscript and modified the figures and tables for improved clarity and readability.
   a. We clarify in two separate sections how the ΦX174+chloramphenicol combination (a) reduces phage-resistance evolution, and (b) biases evolved mutants (reduces diversity of accessible mutations).
   b. We rewrote the results sections to reduce the “whiplash” effect referenced by a reviewer. The first time the model is mentioned, we now clearly outline the assumptions, and describe it in detail. We rewrote how this model can be used to infer the altered mutational target sizes in the phage-antibiotic combinations, compared to phage alone. Then, we introduce the experiment, and the deviation of observations from model predictions, resulting in a need for better models.
   c. We also made it clearer how subsequent (more complicated) models change, and how they can help us to better understand the system. **Figure 3B** now shows the effect size of mutation bias as indicated by model 2, and the potential effect size of the phage-chloramphenicol interaction (which only appears to play a significant role in the phage+1 µg/mL chloramphenicol environment).
   d. Improved methods section: (a) we have clarified how the grayscale values were obtained, and the scales and thresholds used for determining MIC, and (b) provided clearer descriptions of the models and statistical analyses.
