# Supplementary material for: Chloramphenicol and gentamicin reduce the evolution of resistance to phage ΦX174 by suppressing a subset of E. coli LPS mutants
Source: PLoS Biol. 2025 Jan 21;23(1):e3002952. doi: 10.1371/journal.pbio.3002952 (PMC11753469; doi:10.1371/journal.pbio.3002952)
Supplement: Attachment — Submitted filename: Reply_to_reviewers_fourthsubmission.docx [file pbio.3002952.s012.docx]

Dear Editor and reviewers,

We are grateful for the constructive comments we have received. We addressed all the comments and fixed the typos.

------------------------------------------------------------------------
REVIEWERS' COMMENTS

I congratulate the authors on their clear and substantive effort put into revising the manuscript following the previous rounds of comments. I think that this latest version tells a cohesive and compelling story that is nevertheless nuanced and easy to follow. I only have a few very minor comments:

Line 31: I recommend replacing "outer membrane" with "surface". Many sphages use non-outer membrane receptors such as capsule or appendages.

*We changed this.*

Line 117: Replace "surprisingly" with "surprising"

*Fixed, thank you*.

Line 411, Table 2: Check the highlighting of cells in this table. I think there's a mistake in the gene-wise distribution: it's the cells for "Phage" in Models 2 and 3 that should be highlighted instead of the cells for "Phage + 1 ug/ml GM"

*Agreed. We have updated Table 2 in the manuscript and the supplementary .xlsx file.*

Line 426: I suggest you specify here, as you have done in the Methods section, that the "fresh media" in this transfer contained the previously-stated antibiotics as well.

*We changed it to,* “The growth of twelve replicate cultures in each environment was recorded for 24 hours (day 1), after which 2 % of each culture was transferred to fresh media (with the same antibiotic concentrations as day 1) and growth continued for a further 24 hours (day 2).”

Lines 438-439: The statement "but considerably more slowly when gentamicin or chloramphenicol is present" seems inaccurate. Looking at the figure, it seems that this is only true for chloramphenicol but not for gentamicin.

*Agreed. The emergence of resistance with gentamicin occurs at the same time as without antibiotics. We have reworded it and now separately mention the significant difference in growth for 2 µg/mL gentamicin:*

“Further, the course of evolution is affected by the presence of antibiotics: phage resistance emerges rapidly on day 1 when no antibiotic is present, but considerably more slowly when chloramphenicol is present. In 2 µg/mL gentamicin, phage resistance emerges without a delay, yet bacterial growth is significantly inhibited.”

Lines 552-553: I suggest toning down the argument that, because of its narrow host range, ΦX174 is unsuitable for therapeutic purposes. A lot of therapeutic phages have narrow host ranges and have been successfully used in bespoke cases. Perhaps a better description is that ΦX174 is unsuitable for "widespread" therapeutic use?

*We reworded the paragraph to:*

“Understanding the combined effect of phages and antibiotics on bacterial evolution ultimately aims to improve therapeutic outcomes. Unfortunately, ΦX174 itself is currently not a therapeutically relevant phage since it infects only about 0.8 % of 783 *E. coli* human and animal commensal strains (Michel et al., 2010), and no pathogenic strains. However, the host range of ΦX174 can be expanded through evolution experiments (Romeyer Dherbey et al., 2023; Romeyer Dherbey and Bertels, 2024) or genetic engineering (Yehl et al., 2019). Once the limitation of phage entry is overcome, ΦX174 can replicate its DNA and successfully lyse hosts, including various *E. coli* strains and other species such as *S. enterica* and *Enterobacter aerogenes* (Suzuki et al., 1974; Mayr et al., 2005; Orta et al., 2023). Extending the host range of ΦX174 could allow the extensive biological knowledge accumulated over the last few decades to be used to convert ΦX174 into an efficient therapeutic agent, especially when combined with antibiotics to exploit shortened LPS structures (Romeyer Dherbey and Bertels, 2024).”

Line 557: Enterobacter aerogenes is a misnomer. The species has been renamed as Klebsiella aerogenes, check <https://www.ncbi.nlm.nih.gov/pmc/articles/PMC7448666/>

*We changed it, thank you*.

Line 639: There is a typo duplicating the references by Zheng and Rosche and Foster.

*Fixed.*
------------------------------------------------------------------------

Reviewer #2:

I have reviewed two previous versions of this manuscript. The authors have now added some experimental data in liquid culture, that add to the fluctuation assays by allowing to observe some population dynamics in the presence of combination phage+antibiotic treatment, which supports their previous claims. They have also re-analysed their data on MIC effects of phage resistance mutants, with a clearer interpretation provided in Fig 2B.  More generally, the manuscript has been re-written and I believe it reads much better now, with a clearer back and forth discussion between the model and experimental results. I'm happy to recommend it for publication, with only a few remaining comments:


Minor comments

L55 - references for the claim of treatment failure due to phage resistance?

*We have added a reference (Oechslin, 2018) which reviews in detail the emergence of resistance during phage treatment, and how that is related to treatment success or failure.*

L550-562 - I don't think host range considerations are relevant to this paper

*We reworded the paragraph to increase its relevance to the paper:*

“Understanding the combined effect of phages and antibiotics on bacterial evolution ultimately aims to improve therapeutic outcomes. Unfortunately, ΦX174 itself is currently not a therapeutically relevant phage since it infects only about 0.8 % of 783 *E. coli* human and animal commensal strains (Michel et al., 2010), and no pathogenic strains. However, the host range of ΦX174 can be expanded through evolution experiments (Romeyer Dherbey et al., 2023; Romeyer Dherbey and Bertels, 2024) or genetic engineering (Yehl et al., 2019). Once the limitation of phage entry is overcome, ΦX174 can replicate its DNA and successfully lyse hosts, including various *E. coli* strains and other species such as *S. enterica* and *Enterobacter aerogenes* (Suzuki et al., 1974; Mayr et al., 2005; Orta et al., 2023). Extending the host range of ΦX174 could allow the extensive biological knowledge accumulated over the last few decades to be used to convert ΦX174 into an efficient therapeutic agent, especially when combined with antibiotics to exploit shortened LPS structures (Romeyer Dherbey and Bertels, 2024).”

------------------------------------------------------------------------

EDITORIAL REQUESTS

a) We routinely suggest changes to titles to ensure maximum accessibility for a broad, non-specialist readership, and to ensure they reflect the contents of the paper. In this case, we would suggest a minor edit to the title, as follows. Please ensure you change both the manuscript file and the online submission system, as they need to match for final acceptance:
"Chloramphenicol and gentamicin reduce the evolution of resistance to phage ΦX174 by suppressing a subset of E. coli LPS mutants"

*Accepted and changed.*

b) Please provide the grant number in your financial statement.

*We do not have a grant number for any of the grants we were funded by.*

c) We do not have a count limit. Please include the supplementary notes and references in the main text.

*We included the supplementary notes, and supplementary references separate from the main references.*

d) You may be aware of the PLOS Data Policy, which requires that all data be made available without restriction: <http://journals.plos.org/plosbiology/s/data-availability>. For more information, please also see this editorial: <http://dx.doi.org/10.1371/journal.pbio.1001797>
Please supply the numerical values either in the a supplementary file or as a permanent DOI’d deposition for the following figures and tables:

Figure 2AB, 3BC, 4AB, 5ABC, 6BC , S1, S2, S3AB, S4, S5A-E, and Tables 2, S1

*We have included all data points used to make the Figures and Tables.*

e) Please cite the location of the data clearly in all relevant main and supplementary Figure legends, e.g. “The data underlying this Figure can be found in S1 Data” or “The data underlying this Figure can be found in [https://doi.org/10.5281/zenodo.XXXXX”](https://doi.org/10.5281/zenodo.XXXXX)

*We now mention the location of the datasets in the Figure or Table caption, as well as in the Data Availability section.*

f) Please ensure that your Data Statement in the submission system accurately describes where your data can be found and is in final format, as it will be published as written there.

*We updated the Data Availability statement*.

g) Per journal policy, if you have generated any custom code during the course of this investigation, please make it available without restrictions upon publication. Please ensure that the code is sufficiently well documented and reusable, and that your Data Statement in the Editorial Manager submission system accurately describes where your code can be found.

*The code used to calculate chi-squared test values and perform bootstrapping iterations has been uploaded to Edmond (*[*https://doi.org/10.17617/3.PIDVUT*](https://doi.org/10.17617/3.PIDVUT)*) along with its input file, and the Data availability statement has been updated to include it.*
